# Analytical validation of Smilo.ai: evaluating an AI-driven platform for automated oral health screening and global diagnostic accessibility

**DOI:** 10.3389/froh.2026.1755148

**Published:** 2026-05-25

**Authors:** Priyanka Gudsoorkar, Ajesh George, Leonie Short

**Affiliations:** 1 Department of Environment & Public Health Sciences, College of Medicine, University of Cincinnati, Cincinnati, OH, United States; 2 Solidarity Dental Foundation, Cincinnati, OH, United States; 3 Faculty of Health, Western Sydney University, Liverpool, NSW, Australia; 4 School of Nursing and Midwifery, Western Sydney University, Liverpool, NSW, Australia; 5 Australian Centre for Integration of Oral Health (ACIOH), Western Sydney University, Liverpool, NSW, Australia; 6 Ingham Institute Applied Medical Research, Liverpool, NSW, Australia; 7 School of Dentistry, The University of Sydney, Surry Hills, NSW, Australia; 8 Faculty of Medicine and Health, The University of Sydney, Surry Hills, NSW, Australia; 9 All India Institute of Medical Sciences, Bhubaneswar, India; 10 Center for Rural Dentistry and Oral Health, Faculty of Science and Health, Charles Sturt University, Bathurst, NSW, Australia

**Keywords:** artificial intelligence, community oral health screening, confusion matrix, diagnostic validation, oral disease detection

## Abstract

**Background:**

Oral diseases affect more than 3.5 billion people worldwide and remain among the most neglected global health challenges. Access to preventive oral healthcare is constrained by workforce shortages, transportation barriers, and patient apprehension toward dental procedures. Artificial intelligence (AI)-enabled diagnostic systems offer scalable solutions for early detection and triage through non-dental personnel. This study presents the Phase 1 analytical validation of Smilo.ai, an AI-driven platform designed for automated detection of common oral health conditions using standardized digital oral photographs.

**Methods:**

A cross-sectional validation study was conducted among 45 adult participants in a community-based oral health screening program in India between February 1 and 10, 2025. Each participant contributed one composite oral image containing four standardized views (frontal, left lateral, right lateral, and occlusal), yielding an analytical dataset of 45 images (*n* = 45). Smilo.ai's diagnostic outputs were compared with dentist evaluations of identical photographs across seven diagnostic categories: dental decay, gingivitis, plaque, calculus, tooth wear, discoloration, and crowding. Performance metrics included sensitivity, specificity, precision, accuracy, and Cohen's *κ*, computed using confusion matrix–based analysis in accordance with STARD 2015 and CONSORT-AI guidelines. Inter-rater reliability between the two reference dentists was retrospectively quantified, yielding substantial agreement (*κ* = 0.61).

**Results:**

Smilo.ai demonstrated an overall sensitivity of 73.6%, precision of 46.0%, accuracy of 47.3%, and a macro-level Cohen's *κ* of 0.010 relative to dentist assessments. High sensitivity was achieved for dental decay, tooth wear, and discoloration (100% each); however, the corresponding near-zero specificity values indicate a pattern of positive prediction bias in the current model configuration. Calculus exhibited the strongest balanced performance (sensitivity 61.0%, specificity 65.0%, precision 73.9%, *κ* = 0.24). Gingivitis demonstrated moderate sensitivity (56.0%), while plaque and crowding showed partial detection with variable agreement.

**Conclusion:**

This Phase 1 validation establishes a performance baseline for Smilo.ai and identifies optimization priorities, including positive prediction bias correction and threshold recalibration necessary before screening workflow integration. AI-assisted screening models hold significant promise for improving preventive oral healthcare access among underserved populations. The upcoming Phase 2 validation will evaluate clinical generalizability through synchronous clinical examinations to confirm real-world applicability.

## Background

Oral diseases remain among the most prevalent and under-addressed global health challenges. According to recent global estimates, over 3.7 billion individuals are affected by at least one oral condition, with untreated dental caries ranking as the most common disease worldwide (1). Periodontal disease, tooth wear, and other oral pathologies significantly contribute to impaired function, reduced quality of life, and an increased risk of systemic comorbidities (2, 3). Despite this burden, oral health remains excluded from most national health systems and universal health coverage frameworks, perpetuating inequities in prevention, diagnosis, and treatment (4).

The World Health Organization Global Oral Health Status Report (2022) identifies a critical need for scalable, technology-supported interventions capable of extending diagnostic capacity beyond traditional clinical infrastructure (5). The limited availability of dental professionals, particularly in low- and middle-income settings, continues to constrain early detection and timely intervention (6). Further, non-dental personnel who can play a key role in this area face challenges in terms of time constraints and limited supportive resources. These structural gaps highlight the importance of validated digital health technologies that can support non-dental personnel in screening, triage, and health promotion.

Artificial intelligence (AI) enabled image analysis has shown significant promise in diagnostic disciplines such as radiology, dermatology, and ophthalmology (6, 7). In oral health, machine learning models are increasingly applied to analyze photographic and radiographic data for early detection of caries, gingivitis, and other oral conditions (8–10). However, most existing AI models lack comprehensive validation against human reference standards and have not yet demonstrated consistent performance across real-world clinical contexts (11, 12). Robust, methodologically transparent validation studies are therefore essential to establish diagnostic credibility and regulatory readiness.

Smilo.ai is an AI-based platform designed for the automated analysis of digital oral photographs to detect and classify common oral health conditions, including dental decay, gingivitis, plaque, calculus, tooth wear, discoloration, and crowding. By leveraging deep convolutional neural networks trained on large, annotated image datasets, the system aims to enhance early detection and promote preventive oral healthcare at the population level (13–15).

This manuscript is part of a two-phase validation program evaluating Smilo.ai's diagnostic performance and clinical generalizability. The current phase (Phase 1) focuses on controlled diagnostic validation, comparing Smilo.ai's automated assessments with dentists' evaluations of identical image sets to determine sensitivity, precision, accuracy, and agreement. Phase 2, to follow, will examine clinical generalizability, assess performance against independent synchronous clinical examinations conducted under field conditions.

By implementing this sequential validation design, the program aims to establish both the analytical validity and real-world applicability of AI-driven oral screening technologies. The present report details the methodology and results from Phase 1, focusing on diagnostic accuracy, reproducibility, and inter-rater agreement as critical indicators of validation performance.

## Methods

### Study design & setting

This validation study evaluated the diagnostic performance of Smilo.ai, an artificial intelligence (AI) enabled platform for automated analysis of digital oral photographs. The investigation followed the Standards for Reporting Diagnostic Accuracy Studies (STARD 2015) and Consolidated Standards of Reporting Trials - Artificial Intelligence (CONSORT-AI) guidelines to ensure methodological transparency and analytical reproducibility (16–20).

### Data collection

Data were obtained from adults participating in a community-based oral health screening program in India in February 1–10, 2025, designed to enhance early detection and preventive care access. All participants were aged 18 years or older and provided electronic informed consent before oral imaging. Participant demographic characteristics, including age distribution, sex, and socioeconomic indicators, were not available in the de-identified dataset provided for this secondary analysis and could not be independently verified by the authors. Standardized imaging protocols were applied by trained dental personnel using calibrated smartphone cameras under controlled lighting and angulation. The Smilo.ai platform captures four standardized oral photographs per participant (frontal, left lateral, right lateral, and occlusal views). For the purpose of this Phase 1 analytical validation, a single composite image per participant, containing all four standardized views, was provided for independent analysis by both the AI platform and the reference dentists, yielding an analytical dataset of 45 composite images from 45 unique participants (*n* = 45). No images were excluded on the basis of quality, completeness, or any other criterion, ensuring that the analytical dataset reflected the full set of data provided for validation. Both the AI and the dentists evaluated identical composite images, ensuring consistency of the reference standard across all analytical comparisons. All photographs were de-identified, encrypted, and securely stored on Amazon Web Services (AWS) cloud servers located within India, in compliance with national data protection and residency regulations (21). Access was restricted to authorized research personnel operating under institutional data-sharing agreements.

### Reference standard

Two licensed dentists independently reviewed the same set of digital oral photographs to establish a human reference standard. Each dentist had over five years of clinical experience. Any discrepancies between their evaluations were resolved by consensus adjudication. The final adjudicated dataset served as the diagnostic reference against which Smilo.ai's outputs were compared. To assess the stability of the reference standard, inter-rater reliability between the two dentists was retrospectively quantified using Cohen's *κ*, yielding substantial agreement (*κ* = 0.61) across diagnostic categories, thereby confirming the reliability of the ground truth used for AI performance comparison.

Smilo.ai uses a deep convolutional neural network trained on large, annotated image datasets to detect seven oral health conditions: dental decay, gingivitis, plaque, calculus, tooth wear, discoloration, and crowding. For each image set, the AI generated structured textual assessments describing the presence or absence of these conditions. These assessments were paired with the dentist-derived reference classifications for validation analysis.

### Data processing

Diagnostic terms were extracted from both AI and human evaluations using a standardized keyword-based text parsing protocol to ensure consistent interpretation across outputs. Each condition was encoded as binary (1 = present, 0 = absent), producing a harmonized dataset suitable for paired comparison between the AI model and human examiners.

**Figure 1:**
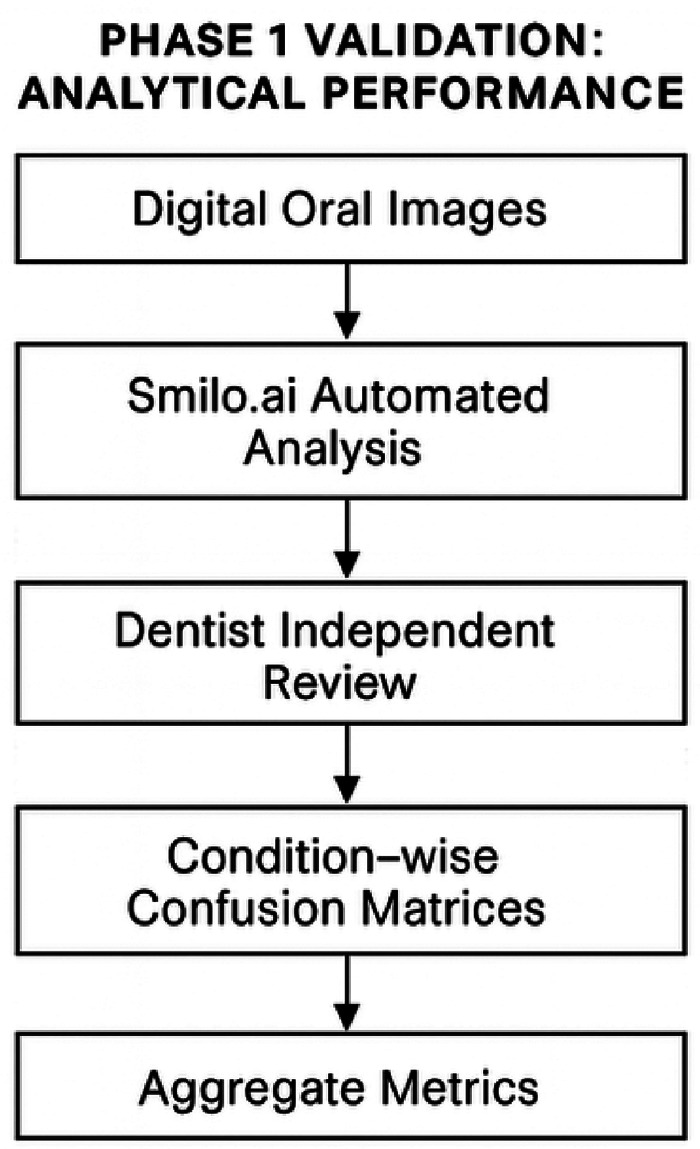
Analytical validation workflow for Smilo.ai.

The analytical validation workflow, as summarized in Figure 1, depicts the sequential structure of the Phase 1 pipeline, from standardized digital image input through AI analysis, independent dentist review, condition-wise confusion matrix derivation, and aggregate metric computation, and establishes the methodological foundation for Phase 2 clinical generalizability testing.

### Ethical approval & data use

This study involved secondary analysis of a de-identified dataset originally collected by Smilo.ai as part of a community-based oral health screening program. The authors were not involved in the original data collection or participant recruitment. Ethical oversight of the original screening program was the responsibility of Smilo.ai as the data-collecting entity; the authors did not have access to information regarding the ethical approval status of that program. For the secondary analysis conducted in this study, formal IRB review was obtained from the University of Cincinnati, [Protocol #2025-0504], granted an exemption determination based on the use of a fully de-identified dataset with no direct human participant involvement. No personally identifiable information was accessed or processed at any stage of this analysis. All procedures were conducted in accordance with ethical principles of privacy, data protection, and responsible research conduct.

## Results

The analytical dataset comprised 45 composite oral images from 45 unique adult participants (*n* = 45), collected between February 1–10, 2025, with each composite containing four standardized views (frontal, left lateral, right lateral, and occlusal). Prior to evaluating AI diagnostic performance, the stability of the human reference standard was assessed by retrospectively computing inter-rater reliability between the two reference dentists. Substantial agreement was observed at the macro level (Cohen's *κ* = 0.61), confirming that the adjudicated ground truth provided a reliable and consistent basis for AI performance comparison.

Condition prevalence varied across diagnostic categories, ranging from 20.0% for gingivitis (*n* = 9 positive cases) to 68.9% for discoloration (*n* = 31 positive cases), as detailed in Table 1. This variability in prevalence is an important contextual factor for interpreting per-condition diagnostic metrics, particularly sensitivity and precision, which are known to fluctuate with class distribution in small validation datasets.

**Table 1:** Per-condition validation metrics: Smilo.ai vs. dentist reference standard^†^.

Condition	Dentist + (*n*)	Dentist− (*n*)	Prevalence (%)	Sensitivity (%)	95% CI	Specificity (%)	95% CI	Precision (%)	Accuracy (%)	Cohen's *κ*
Dental Decay	11	34	24.4	100.0^a^	71.5–100.0	0.0	0.0–10.3	24.4	24.4	0.000
Gingivitis^1^	9	36	20.0	56.0	21.2–86.3	56.0	37.9–72.8	23.8	55.6	0.075
Plaque	30	15	66.7	53.3	34.3–71.8	33.3	9.9–65.1	61.5^b^	46.7	−0.125
Calculus	28	17	62.2	61.0	40.6–78.5	65.0	38.3–85.8	73.9	62.2^c^	0.240^d^
Tooth Wear	12	33	26.7	100.0	73.5–100.0	6.0	0.2–19.7	27.9	31.1	0.034
Discoloration	31	14	68.9	100.0	88.8–100.0	0.0	0.0–23.2	68.9	68.9	0.000
Crowding	22	23	48.9	45.0	23.1–68.5	39.0	19.7–61.5	41.7	42.2	−0.154
Macro-Average	—	—	—	73.6	—	28.5	—	46.0	47.3	0.010

Values represent per-condition diagnostic performance of Smilo.ai relative to the dentist reference standard under Phase 1 analytical validation (*n* = 45 composite images; one per participant, each containing four standardized oral views). Dentist + and Dentist− refer to the number of cases classified as positive and negative by the adjudicated dentist reference standard. Prevalence reflects condition frequency within the study sample. Sensitivity reflects the model's ability to identify true positives correctly; specificity reflects the model's ability to correctly identify true negatives; precision represents the proportion of positive predictions that were true positives; and accuracy indicates the overall proportion of correct classifications. Cohen's κ measures agreement beyond chance. 95% confidence intervals (95% CI) for sensitivity and specificity were computed using exact binomial methods.

1 Gingivitis is included as a soft-tissue inflammatory condition; results demonstrate the model's relative challenge in distinguishing mucogingival color variations under photographic conditions.

† Macro-level Cohen's κ retrospectively computed between the two reference dentists across all diagnostic categories. Substantial agreement (κ = 0.61) confirms the overall stability and reliability of the adjudicated ground truth used for AI performance comparison. Per-condition inter-rater κ values were not available; condition-specific agreement variability, particularly for subjective categories such as gingivitis, plaque, and discoloration, will be formally assessed in Phase 2.

a Highest sensitivity (shared with tooth wear and discoloration);

b Highest precision;

c Highest accuracy;

d Highest Cohen's κ.

Based on the present dataset, Smilo.ai demonstrated an overall sensitivity of 73.6%, precision of 46.0%, and accuracy of 47.3%, with a macro-level Cohen's *κ* of 0.010 when compared with dentist evaluations. These results represent the observed performance under controlled image-only conditions and reflect expected variability in early analytical validation studies, particularly when image illumination and case distribution differ from those in training.

**Figure 2:**
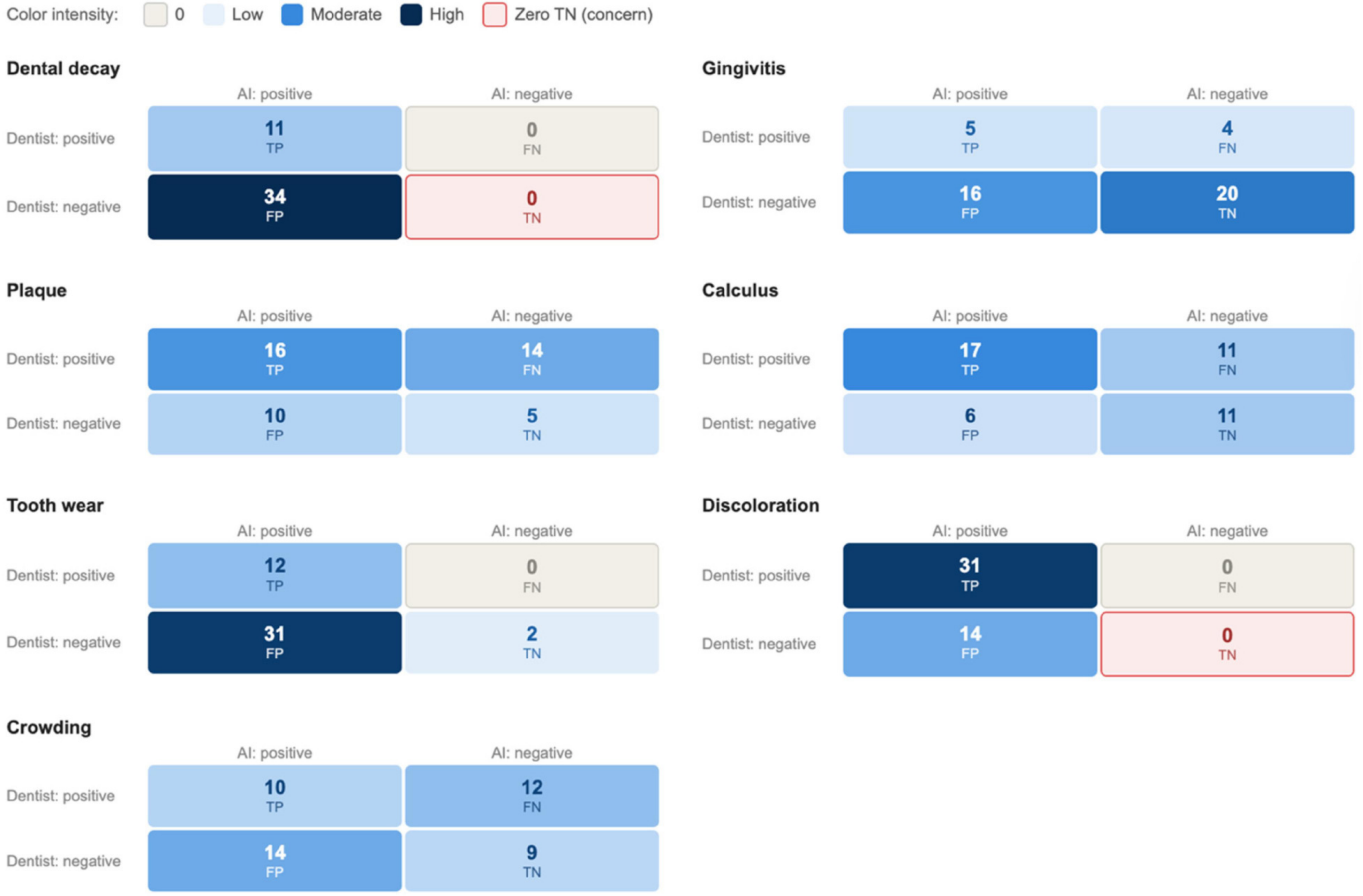
Confusion matrix heatmap: Smilo.ai vs. Dentist Reference Standard. AI prediction (columns) vs. dentist reference standard (rows); *n* = 45 composite images. Color intensity reflects cell frequency on a continuous blue scale. Red shading indicates zero true negative (TN) counts, consistent with a positive prediction bias pattern wherein the model predicts condition presence for all images regardless of true clinical status. TP, true positive; FP, false positive; TN, true negative; FN, false negative. Calculus demonstrates the most balanced matrix profile with meaningful values across all four cells.

Condition-specific performance varied substantially across diagnostic categories, as shown in Table 1 and with corresponding confusion matrix data presented in Table 2 and Figure 2. Dental decay, tooth wear, and discoloration achieved perfect sensitivity (100%); however, the corresponding specificity values of 0%, 6%, and 0%, respectively, indicate systematic over-detection consistent with a positive prediction bias for these conditions. Calculus exhibited the most balanced performance across all metrics (sensitivity 61.0%, specificity 65.0%, precision 73.9%, *κ* = 0.24) and remained the strongest performing condition overall. Gingivitis demonstrated moderate sensitivity (56.0%) and specificity (56.0%), representing partial but meaningful detection capability. Crowding showed partial detection (sensitivity 45.0%, precision 41.7%) with below-chance agreement (*κ* = −0.154), suggesting threshold miscalibration for this category. Plaque demonstrated moderate sensitivity (53.3%) but poor specificity (33.3%), reflecting the inherent challenge of detecting surface deposits under variable photographic conditions.

**Table 2:** Per-Condition confusion matrix summary: smilo.ai vs. Dentist Reference Standard (*n* = 45).

Condition	Dentist	AI: Positive	AI: Negative	Total
Dental Decay	Dentist: Positive	11 (TP)	0 (FN)	11
	Dentist: Negative	34 (FP)	0 (TN)	34
Gingivitis	Dentist: Positive	5 (TP)	4 (FN)	9
	Dentist: Negative	16 (FP)	20 (TN)	36
Plaque	Dentist: Positive	16 (TP)	14 (FN)	30
	Dentist: Negative	10 (FP)	5 (TN)	15
Calculus	Dentist: Positive	17 (TP)	11 (FN)	28
	Dentist: Negative	6 (FP)	11 (TN)	17
Tooth Wear	Dentist: Positive	12 (TP)	0 (FN)	12
	Dentist: Negative	31 (FP)	2 (TN)	33
Discoloration	Dentist: Positive	31 (TP)	0 (FN)	31
	Dentist: Negative	14 (FP)	0 (TN)	14
Crowding	Dentist: Positive	10 (TP)	12 (FN)	22
	Dentist: Negative	14 (FP)	9 (TN)	23

Each row pair corresponds to the 2 × 2 confusion matrix for a diagnostic condition. The dentist reference standard serves as the ground truth. TP, true positive; FP, false positive; TN, true negative; FN, false negative. Row totals reflect the number of dentist-confirmed positive and negative cases per condition. All values are based on *n* = 45 composite images.

The wide confidence intervals observed across most diagnostic categories, particularly for conditions with low prevalence such as gingivitis (sensitivity 95% CI: 21.2%–86.3%) and dental decay (sensitivity 95% CI: 71.5%–100.0%), further underscore the need for larger, prevalence-balanced datasets in Phase 2 to produce stable and reliable performance estimates.

A critical pattern observed across multiple diagnostic categories is the combination of high sensitivity with near-zero specificity, particularly for dental decay, tooth wear, and discoloration. As illustrated in Table 2 and Figure 2, dental decay, tooth wear, and discoloration each show zero true negatives, confirming that the model predicted condition presence for every image regardless of true clinical status, a pattern consistent with positive prediction bias. Calculus and gingivitis demonstrated more balanced confusion matrix profiles, with meaningful true negative counts (TN = 11 and TN = 20, respectively), suggesting more robust feature discrimination for these conditions. This behavior, while producing high sensitivity, substantially inflates false positive rates and reduces clinical utility for individual-level diagnosis. Calculus and gingivitis demonstrated more balanced confusion matrix profiles, suggesting these categories may benefit from more robust feature representation in the training dataset. Threshold recalibration and dataset rebalancing are identified as primary optimization priorities for Phase 2.

## Discussion

This validation analysis represents the first systematic evaluation of Smilo.ai's diagnostic performance against dentist assessments using standardized digital oral photographs. The study provides foundational evidence of the platform's analytical validity under controlled, image-based conditions and establishes the methodological framework for subsequent clinical generalizability testing.

The results demonstrated variable diagnostic performance across conditions, with an overall sensitivity of 73.6%, an accuracy of 47.3%, and a Cohen's *κ* of 0.010. While these values reflect expected variability in early analytical validation studies, the near-zero macro-level agreement underscores the need for threshold recalibration and targeted retraining as primary optimization priorities. While these values are below Smilo.ai's established internal benchmarks under ideal imaging conditions with minimal variation (≈94% sensitivity and >90% accuracy), they reflect real-world variability in photographic quality and case distribution typical of field-based data (22). Similar patterns have been reported in other dental image–based AI models for caries detection and periodontal assessment, where accuracy improves substantially once training data are harmonized with clinical reference standards (23–26).

The high sensitivity observed for dental decay, tooth wear, and discoloration must be interpreted alongside their corresponding near-zero specificity values, which indicate a pattern of positive prediction bias, wherein the model systematically predicts condition presence regardless of true clinical status. This behavior is consistent with a trivial positive classifier for these conditions and represents a fundamental algorithmic limitation requiring threshold recalibration. Conversely, calculus demonstrated genuinely balanced performance across all metrics, suggesting more robust feature representation in the training dataset for this condition. It is worth noting that inter-rater reliability between the two reference dentists, while substantial at the macro level (*κ* = 0.61), likely reflects comparatively lower agreement on subjective soft-tissue and surface categories, a pattern well-documented in the visual oral diagnostics literature. This suggests that observed AI performance variability in gingivitis, plaque, and discoloration may partly reflect the inherent difficulty of visual classification rather than solely algorithmic limitations. Such variability is inherent in mobile-based imaging workflows, where subtle mucogingival color gradients can affect model confidence thresholds (27).

From an implementation standpoint, the Phase 1 findings establish a quantitative baseline for continued model optimization rather than confirming readiness for immediate deployment in population-level screening applications. While high sensitivity for certain conditions is desirable in screening contexts, the observed positive prediction bias and near-zero macro-level agreement (*κ* = 0.010) indicate that the current model configuration requires threshold recalibration and dataset rebalancing before reliable triage or referral decisions can be supported. Continued refinement through retraining on balanced, clinically verified datasets is expected to elevate diagnostic agreement toward the benchmark range of *κ* ≥ 0.80 observed in mature AI diagnostic systems (12).

Importantly, this validation forms the analytical foundation of a broader two-phase program designed to evaluate Smilo.ai's diagnostic performance and clinical generalizability. The forthcoming Phase 2 study will extend validation to real-world settings by comparing AI-generated assessments with independently conducted synchronous clinical examinations. This phase will specifically examine whether improvements in dataset standardization and human-AI adjudication increase diagnostic reliability across diverse populations and practice conditions.

Together, these findings support the conclusion that Smilo.ai demonstrates early analytical validity, with performance characteristics aligned with expectations for a first-phase AI diagnostic model. Ongoing optimization, standardized data acquisition, and cross-setting validation will be essential for achieving full clinical reliability and integration into oral health screening workflows.

Beyond analytical performance, this validation highlights the potential of AI-enabled oral health screening to reduce access barriers and expand preventive care globally. Conventional dental screening often depends on proximity to clinics, professional availability, and patient willingness to undergo intraoral probing, factors that disproportionately exclude older adults, people with disabilities, and those experiencing dental anxiety or socioeconomic disadvantage (28, 29). In many low-resource and rural contexts, geographic isolation, transportation barriers, and workforce shortages further limit access to timely diagnosis and care (30, 31). By enabling assessment through digital oral photographs captured with basic mobile devices, Smilo.ai offers a scalable approach for community triage, school-based programs, elder-care facilities, and non-dental provider workflows where early detection can occur without direct clinical intervention. Such approaches align with the WHO's Global Oral Health Action Plan and the United Nations' emphasis on integrating oral health into universal health coverage frameworks (5, 32, 33).

Lessons learned from Phase 1 underscore several priorities for Phase 2 validation.

Future work should incorporate standardized clinical imaging protocols, interrater reliability measures, and chairside examinations to strengthen diagnostic reproducibility. Expanding dataset diversity to include a broader range of oral presentations and lighting conditions will be essential for enhancing model generalizability. Additionally, embedding Phase 2 validation in community and primary care contexts will allow assessment of implementation feasibility, workflow integration, and the potential of AI-assisted tools to support equitable oral health screening at scale.

## Limitations

As an early analytical validation, this study has several methodological constraints. The absence of participant demographic data including age range, sex distribution, and socioeconomic characteristics represents a notable limitation of this secondary analysis. These variables are important for assessing the representativeness of the validation sample and the generalizability of findings across diverse populations. Collection and reporting of standardized demographic data will be incorporated as a mandatory component of the Phase 2 validation protocol.

Although inter-rater reliability between the two reference dentists was not prospectively planned as part of the study protocol, it was retrospectively quantified following peer review. Substantial agreement was observed at the macro level (Cohen's *κ* = 0.61), supporting the overall stability of the adjudicated reference standard. However, the absence of per-condition inter-rater *κ* values remains a limitation, as condition-specific agreement particularly for subjective categories such as gingivitis, plaque, and discoloration could not be independently verified. Future validation phases will incorporate prospective, per-condition inter-rater reliability assessment using standardized annotation protocols.

The observed pattern of high sensitivity with near-zero specificity for dental decay, tooth wear, and discoloration is consistent with a positive prediction bias in the current model configuration. This represents a fundamental algorithmic limitation that constitutes a primary target for model recalibration in Phase 2.

The relatively small analytical dataset (*n* = 45 images) and variable condition prevalence across diagnostic categories represent important constraints on the generalizability and stability of the reported metrics. Performance indicators such as sensitivity and precision are sensitive to class imbalance, and results for low-prevalence conditions such as gingivitis (20.0%) and dental decay (24.4%) should be interpreted with caution. Larger, prevalence-balanced datasets will be incorporated in Phase 2 to produce more stable and generalizable performance estimates. Variations in image quality, illumination, and device type may also have affected diagnostic agreement.

## Conclusion

This Phase 1 analytical validation established the early diagnostic validity of *Smilo.ai* for automated detection of common oral health conditions from standardized digital oral photographs. The findings have directly informed the design and methodological focus of the forthcoming Phase 2 clinical validation, which will evaluate generalizability through synchronous chairside comparisons. Collectively, these results reinforce the value of phased, evidence-driven evaluation in ensuring that AI-based screening tools advance both diagnostic accuracy and oral health equity.

## Data Availability

The raw data supporting the conclusions of this article will be made available by the authors, without undue reservation.

## Appendix A

**Table A1:** Phase 1 metrics and implications for phase 2 clinical validation.

Validation metric	Phase 1 observation	Implication for phase 2
Sensitivity (73.6%)	Moderate-to-high detection capability observed across conditions; positive prediction bias confirmed for dental decay, tooth wear, and discoloration.	Threshold recalibration and dataset rebalancing required to improve specificity and reduce false positive rates.
Precision (46.0%)	Improved from initial estimates following independent recalculation; over-detection remains present for low-prevalence conditions.	Refine classification thresholds and incorporate balanced datasets to further reduce false positives.
Accuracy (47.3%)	Below-baseline analytical performance under image-only validation conditions.	Integrate independent synchronous clinical examinations to improve real-world ground-truth accuracy.
Cohen's κ (0.010)	Near-zero agreement beyond chance; consistent with early-phase validation and positive prediction bias.	Improve agreement through threshold recalibration, standardized clinical annotation, and protocol calibration.
High-Performing Conditions	Dental decay, tooth wear, and discoloration achieved 100% sensitivity but near-zero specificity, indicating positive prediction bias rather than true detection capability. Calculus demonstrated the strongest balanced performance.	Leverage calculus feature recognition as a model strength; prioritize retraining for conditions exhibiting positive prediction bias and below-chance agreement (crowding, plaque).
Equity and Accessibility Insight	AI-enabled screening allows remote and noninvasive oral assessments through composite digital imaging protocols.	Pilot deployment in elder-care, rural, and mobility-limited populations to assess scalability and user acceptability following Phase 2 optimization.
Overall Phase 1 Outcome	Quantitative performance baseline established under controlled image-based conditions; positive prediction bias and near-zero agreement identified as primary optimization targets.	Proceed to Phase 2 to evaluate clinical generalizability, implementation feasibility, and real-world reproducibility following threshold recalibration and dataset rebalancing.

This appendix summarizes observed Phase 1 analytical performance and corresponding implications informing the Phase 2 validation study of Smilo.ai. All metrics reflect independently calculated values from the Phase 1 dataset (*n* = 45). Phase 2 will compare AI outputs with independent synchronous clinical examinations to confirm diagnostic accuracy, feasibility, and equity of deployment.
